# Sustainable green synthesis of zinc oxide nanoparticles utilizing *Zingiber officinale* peel aqueous extract, characterization, and determination of its anticancer and antimicrobial potential

**DOI:** 10.1371/journal.pone.0334685

**Published:** 2025-11-19

**Authors:** Omaish S. Alqahtani, Uday M. Muddapur, Keerti Kamat, Aparna Shenvi, Ibrahim Ahmed Shaikh, Ibrahim Aljaezi, Bassam S. M. Al Kazman, Mohammed A. Alshamrani, Aejaz Abdullatif Khan, Awad Mohammed Al-Qahtani, Basheerahmed Abdulaziz Mannasaheb, Layikh Ahmed

**Affiliations:** 1 Department of Pharmacognosy, College of Pharmacy, Najran University, Najran, Saudi Arabia; 2 Department of Biotechnology, KLE Technological University, BVB Campus, Vidyanagar, Hubballi, Karnataka India; 3 Department of Pharmacology, College of Pharmacy, Najran University, Najran, Saudi Arabia; 4 Department of Pharmaceutical Sciences, Pharmacy College, Umm AlQura University, Makkah, Saudi Arabia; 5 Department of General Science, Ibn Sina National College for Medical Studies, Jeddah, Saudi Arabia; 6 Department of Family and Community Medicine, College of Medicine, Najran University, Najran, Saudi Arabia; 7 Department of Pharmacy Practice, College of Pharmacy, AlMaarefa University, Ad Diriyah, Riyadh, Saudi Arabia; 8 Department of Pharmaceutical Chemistry, College of Pharmacy, Najran University, Najran, Saudi Arabia; Cairo University, Faculty of Science, EGYPT

## Abstract

This study demonstrates the green synthesis, characterization, and biomedical applications of zinc oxide nanoparticles (ZnONPs) using *Zingiber officinale (Z. officinale)* (ginger) peel extract. Green synthesis offers advantages over conventional methods, including environmental friendliness, cost-effectiveness, and enhanced biocompatibility. Ultraviolet-Visible (UV-Vis) spectroscopy confirmed ZnONPs formation with a peak at 364 nm. Fourier-Transform Infrared (FTIR) spectroscopy analysis revealed characteristic peaks indicating functional groups involved in nanoparticle formation. Scanning Electron Microscope (SEM) analysis showed spherical/agglomerated nanoparticles, and Energy-Dispersive X-ray Spectroscopy (EDS) confirmed 77.7% zinc oxide by mass%. The X-ray Diffraction (XRD) indicated an average particle size of 24.67 nm with distinct crystal orientations. Phytochemical analysis detected alkaloids, saponins, and steroids in the extract. Optimal synthesis occurred at 50–60°C and pH 10, yielding stable ZnONPs. The ZnONPs exhibited significant antibacterial activity against *Staphylococcus aureus (S. aureus), Bacillus subtilis (B. subtilis), Bacillus cereus (B. cereus), Escherichia coli (E.coli), Zymomonas mobilis (Z. mobilis)*, and *Pseudomonas aeruginosa (P. aeruginosa)*, as well as antifungal activity against *Candida albicans (C. albicans)*. The in vitro cytotoxicity study on M.D. Anderson – Metastatic Breast – 231 (MDA-MB-231) breast cancer cells showed a dose-dependent reduction in cell viability [Half-maximal Inhibitory Concentration (IC_50_) = 82.13 µg/mL] with notable morphological changes at higher concentrations. The ZnONPs synthesized from ginger peel extract are innovative, environmentally friendly, and economical. Our findings show that biologically generated ZnONPs are effective antibacterial and antifungal agents against several pathogens. This research uniquely demonstrates the potential of ginger peel, a commonly discarded agro-waste, as a sustainable source for ZnONPs synthesis, highlighting its biotechnological and medicinal applications. The novelty of this study lies in the green synthesis approach using ginger peel and the comprehensive evaluation of its antimicrobial and anticancer properties. Further in-depth studies and optimization are needed to validate their therapeutic efficacy and safety.

## 1. Introduction

Nanoparticles (NPs), defined as particles with at least one dimension smaller than 100 nm, are fundamental building blocks in nanotechnology. While such particles have been used for centuries across various industries, recent advancements in their synthesis and precise manipulation have significantly renewed scientific interest. Nanoscale materials find applications in fields such as materials science, biomedicine, pharmaceuticals, energy, environmental remediation, electronics, magnetics, optoelectronics, and cosmetics [1]. Traditionally, nanoparticles are synthesized using physical or chemical methods, which often involve hazardous chemicals and energy-intensive processes, raising environmental and safety concerns.

ZnONPs, a prominent class of metal oxide nanoparticles, have garnered considerable attention due to their tunable optical and chemical properties. ZnONPs exhibit remarkable photocatalytic and photo-oxidative capabilities, making them effective against a wide range of chemical and biological agents [2]. Their chemical stability, cost-effectiveness, electrical conductivity, piezoelectric behavior, and optical transparency make them ideal for applications in gas sensing, photovoltaics, coatings, polymer composites, pharmaceuticals, lasers, and optoelectronic devices [3]. However, conventional synthesis methods often rely on toxic chemicals, limiting their biocompatibility and environmental sustainability.

The growing emphasis on environmental conservation has driven demand for eco-friendly synthesis processes. Green synthesis has emerged as a promising alternative, offering affordability, simplicity, scalability, and reduced reliance on hazardous chemicals while producing biocompatible and stable nanoparticles [4]. Plant extracts, rich in phytochemicals such as tannins, flavonoids, proteins, alkaloids, and amino acids, serve as reducing and stabilizing agents in green synthesis. These bioactive compounds, extracted using environmentally benign solvents, facilitate the conversion of metal ions into nanoparticles through oxidation reactions [4].

Ginger (*Z. officinale* Roscoe), a rhizome of the *Zingiberaceae* family, is widely used as a spice, vegetable, and condiment in tropical regions [5]. Valued in Indian Ayurvedic medicine for centuries [6], ginger possesses strong antioxidant properties [7] and is cultivated extensively in regions such as Asia, Africa, India, Jamaica, Mexico, and Hawaii [8]. Ginger peel, often discarded as agro-waste during food processing, is rich in polysaccharides and bioactive compounds with anti-inflammatory, anticancer, spasmolytic, and diuretic properties [6]. The phytochemical content of ginger peel, including flavonoids and gingerols, can vary with seasonal and environmental factors, influencing its efficacy in green synthesis [9]. Utilizing ginger peel enhances sustainability due to its year-round availability, economic benefits, and positive environmental impact. Ginger is a perennial crop with consistent production across tropical and subtropical regions, ensuring a steady supply of peel waste from food industries [8].

Despite the potential of ginger peel in green synthesis, current studies often employ standard aqueous extraction methods without optimizing the extraction process to maximize bioactive compound yield [10,11]. For instance, previous work using ginger rhizome extracts focused on varying synthesis conditions like precursor concentration or pH but rarely explores advanced extraction techniques or systematic optimization of extraction parameters such as solvent type, pH, or temperature [10,12]. This lack of optimization limits control over nanoparticle (NP) size, morphology, and stability, which are critical for enhancing biomedical applications like antibacterial, antifungal, and anticancer activities [13]. Consequently, there is a significant research gap in developing optimized extraction protocols for ginger peel to produce ZnONPs with tailored properties for improved performance in biomedical contexts. Economically, repurposing ginger peel, which is typically discarded, reduces waste disposal costs and provides a low-cost raw material for NP synthesis, benefiting both industries and local economies [14]. Environmentally, valorizing this agro-waste minimizes landfill accumulation and greenhouse gas emissions associated with organic waste decomposition, aligning with circular economy principles [15]. These attributes make ginger peel an ideal, sustainable candidate for green synthesis.

Cancer is the second leading cause of death in developed countries worldwide [16]. The most common cause of cancer-related death in both females and males is lung cancer, accounting for 1,380,000 deaths annually. Several factors contribute to the high mortality of lung cancer, including inactivity, smoking, and diet. Breast cancer remains one of the most prevalent malignancies worldwide, particularly affecting women across diverse geographic and socioeconomic contexts. According to the World Cancer Research Fund, breast cancer was the most commonly diagnosed cancer in women globally in 2022, with an estimated 2,296,840 new cases reported [17]. Breast cancer continues to be the most commonly diagnosed cancer among women worldwide in 2025, with its burden rising across both high-income and low- to middle-income countries. According to the Global Cancer Observatory, breast cancer accounted for approximately 2.5 million new cases globally in 2025, marking a steady increase from 2.3 million cases in 2020. These statistics underscore the importance of global collaboration in reducing the burden of breast cancer through early detection, equitable access to care, and robust prevention strategies.

The increasing demand for environmentally sustainable nanomaterials has driven significant interest in green synthesis approaches. Conventional methods for synthesizing ZnONPs often involve hazardous chemicals, raising concerns about biocompatibility and ecological impact. To address this gap, the present study introduces a novel, eco-friendly method for the synthesis of ZnONPs using ginger peel extract, with a particular emphasis on optimizing the extraction process.

The reduction of metal ions into nanoparticles using plant-derived compounds, commonly referred to as phytomining, has emerged as a widely adopted protocol in recent years. Plant extracts serve multifunctional roles as reducing, capping, and stabilizing agents, facilitating nanoparticle formation without the need for toxic reagents. Various plant components, including leaves, stems, roots, shoots, flowers, bark, seeds, and their secondary metabolites, have demonstrated efficacy in nanoparticle biosynthesis with several biomedical applications.

Notably, ZnONPs have shown promising anticancer activity against breast cancer cell lines, such as MDA-MB-231, Michigan Cancer Foundation-7 (MCF-7), and human epithelial breast cancer cell line (T-47D), by inducing apoptosis through reactive oxygen species (ROS) generation and mitochondrial dysfunction [18,19]. Green-synthesized ZnONPs, using plant extracts like *Rhus coriaria* or *Rubus fairholmianus*, have demonstrated selective cytotoxicity against triple-negative (MDA-MB-231) and hormone receptor-positive (MCF-7, T-47D) breast cancer cells via ROS-mediated apoptosis and modulation of apoptotic genes (e.g., BCL2-associated protein X (Bax), B-cell lymphoma 2 (Bcl-2)) [20,21]. However, conventional synthesis methods often rely on toxic chemicals, limiting their biocompatibility and environmental sustainability.

Hence, this study was designed to address this gap by introducing a novel approach of synthesizing biogenic ZnONPs using ginger peel extract, with a focus on optimizing the extraction process. By employing solvent extraction and systematically varying parameters such as pH, temperature, and solvent type, this work enhances the yield of bioactive compounds, resulting in ZnONPs with improved physicochemical properties. The synthesized nanoparticles were characterized, and their synthesis conditions (temperature and pH) were optimized. Furthermore, their antibacterial, antifungal, and anticancer properties were evaluated, demonstrating their potential in biomedical and environmental applications.

## 2. Materials and methods

### 2.1. Harvesting and preparation of bioactive extracts

**Fig 1:**
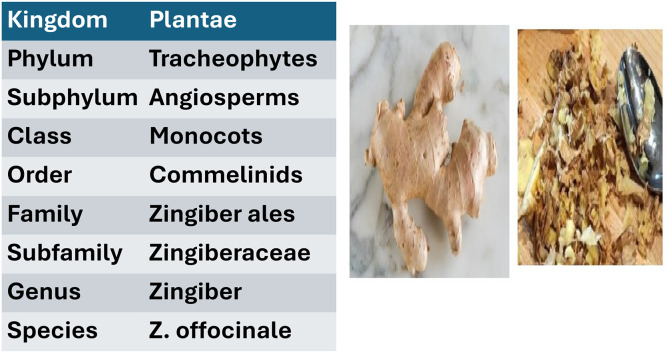
General description and characteristics of *Zingiber officinale.*

Ginger peel (*Z. officinale* R.) was collected randomly from local market vendors during the summer season in Dharwad city, Karnataka, India. The ginger was washed, peeled, and 15 grams of the peel were weighed. The peel, a byproduct of food processing, was obtained fresh to ensure quality. Around 150 mL of distilled water were added to the peel. The mixture was boiled for 15 minutes followed by filtration using a Whatman filter paper. The filtered extract was allowed to cool at room temperature and then stored for further use (Fig 1).

### 2.2. Zinc Oxide Nanoparticle (ZnONPs) synthesis

**Fig 2:**
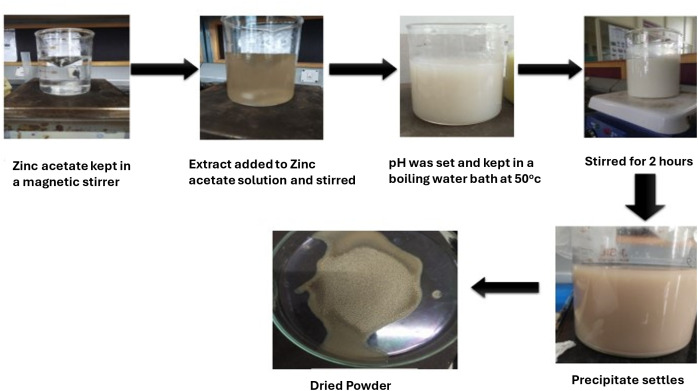
Different steps involved in the synthesis of zinc oxide nanoparticles from *Zingiber officinale* peel extract.

To prepare a 0.1 M zinc acetate solution, 6.58 g of zinc acetate dihydrate was accurately weighed and dissolved in approximately 300 mL of distilled water. When the zinc acetate had dissolved completely, 60 mL of the extract was added incrementally to reach 240 mL while stirring constantly. The pH was adjusted to 10 by freshly prepared 2M NaOH after the extract had been added for one hour. The solution was maintained in a water bath at 50 °C for one hour, followed by continuous stirring for an additional two hours. Upon completion, a white crystalline precipitate was observed to settle. The supernatant was carefully discarded, and the precipitate was collected and dried in a hot air oven at 50 °C. The resulting powder was then stored for further analysis and tests (Fig 2).

### 2.3. Analytical techniques used for the characterization of ZnONPs

#### 2.3.1. Ultraviolet-Visible (UV-visible) spectroscopy analysis.

Primary characterization of the nanomaterials may be achieved with great efficiency and dependability using UV-Vis spectroscopy. UV-Vis is quick, simple, straightforward, sensitive, specific for different types of NPs, requires little time for estimation, and, most importantly, doesn’t require calibration for particle characterisation of colloidal suspensions [22]. In the current study, a spectrophotometer (UV 9600A, Shimadzu Instruments Co., Ltd., Kyoto, Japan), was employed to measure the absorbance of ZnONPs powder synthesized from the *Z. officinale* peel extract and subsequently dispersed in distilled water, using distilled water as the blank reference.

#### 2.3.2. Fourier-Transform Infrared Spectroscopy (FTIR)-Based Analysis of the Prepared ZnONPs.

FTIR is a widely employed modality for analysing chemical interface of nanoparticles. It offers valuable insights into the biomolecular constituents mediating reduction and stabilization processes during nanoparticle synthesis. The presence and type of functional groups and chemical bonds can be inferred from the intensity, shape, and position of absorption bands in the FTIR spectrum. The successful formation of ZnONPs is typically indicated by a characteristic Zn–O bond absorption peak observed in the fingerprint region of the spectrum [23]. In this study, FTIR analysis was carried out using a Thermo Scientific Nicolet iSTM50 FTIR Spectrometer.

#### 2.3.3. Scanning Electron Microscope (SEM) with Energy Dispersive Spectroscopy (EDS) analysis.

The elemental composition and surface morphology are examined, respectively. SEM offers high-resolution images of the material’s morphology and can provide information on the forms, sizes, and surface morphology of nanoparticles [24]. SEM determines the secondary electrons released upon interaction between the sample and the impinging electron beam [25]. The elemental compositions of the material under test are characterized and quantified using EDS [24]. The analysis was performed using Scanning Electron Microscope manufactured by JEOL model number JSM-IT500L.

#### 2.3.4. X-ray Diffraction (XRD) analysis.

The size and shape of the unit cells, as well as the degree of translational symmetry, which is deduced from the peak positions, are crucial details revealed by the XRD analytical methods used to identify the metallic nature of nanoparticles. With higher intensities signifying a greater presence of atoms, these techniques also provide information about the electron density inside the unit cells. The presence of nanoparticles in the synthesized material is indicated by the distinctive broadening of the XRD peaks. A Full Width at Half Maximum (FWHM) analysis model was used to extract information on the maximum intensity, peak position, and peak width [26]. The size of the crystallite is correlated with peak broadness in the XRD pattern. The average crystallite diameter was determined using the Scherrer equation [27].

#### 2.3.5. Preliminary phytochemicals screening.

A preliminary screening of phytochemicals was performed to detect bioactive secondary metabolites present within the ginger peel extract. Various phytochemicals like alkaloids, flavonoids, saponins, steroids, etc. were tested [28–32].

#### 2.3.6. Modulation of nanoparticle synthesis by effect of temperature.

The temperature of the solution can regulate the size of biosynthesized nanoparticles; a rise in temperature causes the ZnONPs to enlarge. The temperature-dependent rise in nanoparticle size implies that the rate of metal ion reduction increases with a subsequent increase in temperature. Reaction kinetics are known to accelerate at higher temperatures. However, due to the high kinetics involved, controlling the growth phase of the crystallization process during reactions can be a challenging process [33]. To examine the effect of temperature, UV spectra at 30°C to 60°C were recorded.

#### 2.3.7. Effect of pH on the biosynthesis of ZnONPs.

The influence of pH on the biosynthesis of ZnONPs was systematically evaluated by adjusting the reaction medium to various pH conditions. The synthesis is impacted by the solution’s changing pH levels. The size and texture of biosynthesized nanoparticles can be significantly influenced by the pH level. Additionally, both the shape and size of the obtained nanoparticles have been managed by adjusting the pH level [34]. The pH of the solution plays a critical role in determining the physicochemical properties of the synthesized nanoparticles. It is observed that the shape, size, and surface morphology of ZnONPs is significantly affected by pH variation [34]. At neutral pH (≈7), the nanoparticles exhibit relatively uniform morphology with minimal surface alterations. In contrast, under alkaline conditions (pH > 7), the elevated concentration of hydroxide ions (OH⁻) facilitates enhanced nucleation and crystallization processes, leading to the formation of smaller, well-defined ZnONPs. These findings underscore the importance of pH optimization in tailoring nanoparticle characteristics for specific biomedical applications [35]. In the current study, to investigate the effect of pH on synthesis, the pH was adjusted to 6, 8, and 11 using 1M HCl and 2M NaOH. The absorbance was measured using a UV-Vis spectrophotometer in the range of 200–800 nm.

### 2.4. Biomedical applications

#### 2.4.1. Anticancer activity.

In the present study the effect of synthesized ZnONPs on the viability of MDAMB-231 cells was determined based on the 3-(4,5-dimethylthiazol-2-yl)-2,5-diphenyltetrazolium bromide (MTT) calorimetric assay [36]. Initially the monolayer cell culture was trypsinized and the cell count was adjusted to 1.0 X 105cells/mL using Dulbecco’s Modified Eagle Medium (DMEM) containing 10% Fetal Bovine Serum (FBS) and transferred to 96-well µL plates for growth (Falcon, Becton– Dickinson, Franklin Lakes, NJ, USA). After 24 h of incubation, the old media was removed and replaced with fresh media along with different concentrations (20 µg/mL, 40 µg/mL, 60 µg/mL, 80 µg/mL and 100 µg/mL) of test sample, the assay was terminated after 24 h, the medium was removed and 200 µL of Dimethyl sulfoxide (DMSO) was added and the amount of formazan formed was measured at 595 nm on a Model 680 Micro plate reader (Bio-Rad Laboratories, Inc., Hercules, CA, USA). The percentage growth inhibition was calculated using the following formula and concentration of test drug needed to inhibit cell growth by 50% (IC_50_) was calculated from the dose-response curves for each cell line. This assay is based on the reduction of MTT by the mitochondrial dehydrogenase of intact cells to a purple formazan product [37].


𝐈𝐧𝐡𝐢𝐛𝐢𝐭𝐢𝐨𝐧 𝐏𝐞𝐫𝐜𝐞𝐧𝐭𝐚𝐠𝐞 =𝐎𝐃 𝐨𝐟 𝐓𝐞𝐬𝐭 𝐬𝐚𝐦𝐩𝐥𝐞÷𝐎𝐃 𝐨𝐟 𝐜𝐨𝐧𝐭𝐫𝐨𝐥×100

#### 2.4.2. Well diffusion-based screening of antibacterial and antifungal properties.

Nutrient agar plates were prepared under sterile conditions. Bacterial cultures grown in nutrient broth for 24 h were used for inoculation. Wells of approximately 6–7 mm in diameter were created in the agar plates, and 100 µg of ZnONPs were introduced into each well. After being diluted to 0.1 mL, each bacterial culture was applied to both positive and negative controls. The clearance zone of inhibition (mm) was measured and noted following a 24-h incubation period at 37 °C [38,39]. Ampicillin (25 µg/mL) was used as a standard antibiotic for comparison. The zone of inhibition was used to assess the antibacterial action [39].

#### 2.4.3. Antifungal activity against *Candida albicans.*

The effectiveness of the ZnONPs against the *C. albicans* fungal pathogen was assessed using a conventional disc diffusion technique [40]. The standard antifungal control used was fluconazole [41]. For antimicrobial assessment, the inoculum was uniformly spread across the surface of agar plates containing potato dextrose medium. Sterile discs were prepared using Whatman filter paper and soaked in the test solution (100 µg/mL) for 3–4 h. The impregnated discs were then aseptically placed on the inoculated agar surface using sterilized forceps. The plates were incubated at the appropriate temperature (typically 28–30 °C for fungi) for 24 h to observe the zone of inhibition and evaluate antifungal activity.

### 2.5. Statistical analysis

With MINITAB statistical software version 18.1 (MINITAB Inc., State College, PA, USA), the outcomes were subjected to analysis of variance (ANOVA) and compared with the Tukey test at a significance level of 5%.

## 3. Results and discussion

### 3.1. Profiling of Synthesized ZnONPs

#### 3.1.1. UV visible spectroscopy analysis.

**Fig 3:**
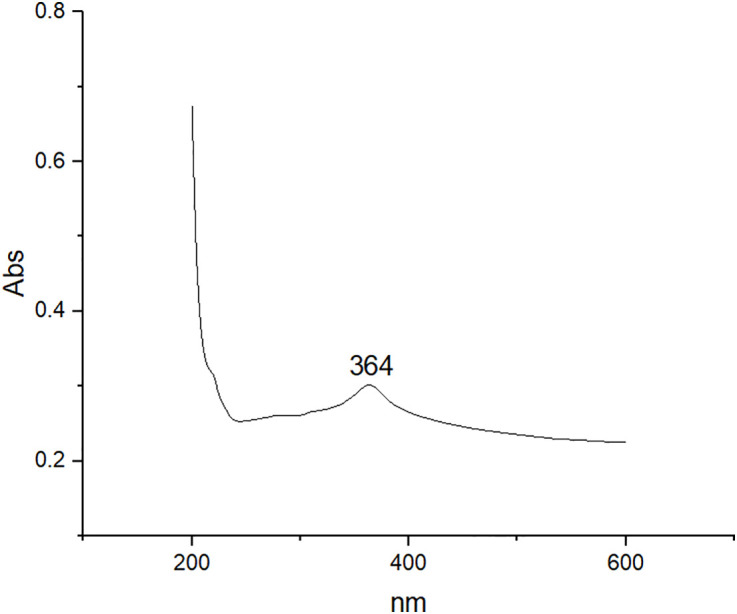
Ultraviolet-Visible (UV-Vis) absorption spectrum of zinc oxide nanoparticles (ZnONPs) synthesized using ginger peel extract. This figure shows the UV-Vis spectrum of biosynthesized ZnONPs, with a prominent absorption peak at 364 nm. This peak corresponds to the characteristic absorption edge and bandgap energy of ZnONPs, which are key indicators of their optical properties. The presence of this peak confirms the successful formation of ZnONPs and reflects their nanoscale dimensions.

The peak appearance at 364 nm in the UV–Vis spectrum of ZnONPs (Fig 3) reveals their unique optical characteristics. This wavelength corresponds to the absorption edge and bandgap energy of ZnONPs, offering crucial insights into their size, structure, and electronic properties. An edge absorption peak at 368 nm of synthesized ZnONPs was reported [30].

#### 3.1.2. FTIR profiling of zinc oxide nanoparticles.

**Fig 4:**
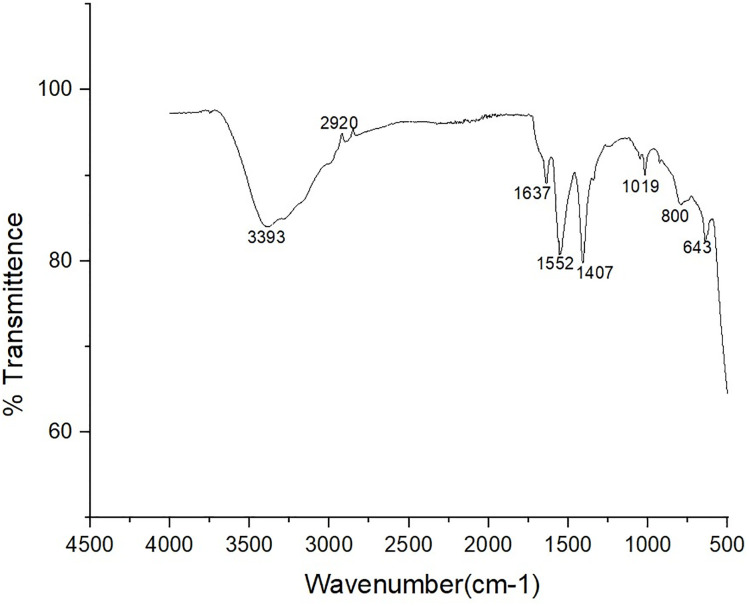
Fourier Transform Infrared (FTIR) spectrum of zinc oxide nanoparticles (ZnONPs) synthesized using ginger peel extract. This figure displays the FTIR spectrum used to identify functional groups involved in the formation and stabilization of ZnONPs. The spectrum shows characteristic absorption bands at 3393 cm ⁻ ¹ (O–H stretching in alcohols and phenols), 2920 cm ⁻ ¹ (C–H stretching in alkanes or methyl groups), 1637 cm ⁻ ¹ (C = O stretching in ketones, esters, or aldehydes), and 1552 cm ⁻ ¹ (N–H bending in primary amines or amides). Additional peaks include 1407 cm ⁻ ¹ (C–H bending in methyl groups), 1019 cm ⁻ ¹ (C–O stretching in ethers or secondary alcohols), 800 cm ⁻ ¹ (aromatic C–H out-of-plane bending), and 643 cm ⁻ ¹ (C–H bending in substituted benzene rings or alkenes). These functional groups originate from bioactive compounds in the ginger peel extract and play a role in reducing and stabilizing the nanoparticles. The presence of Zn–O bonds is confirmed by peaks in the 400–700 cm ⁻ ¹ region and at 1018 cm ⁻ ¹, validating the formation of ZnONPs.

ZnONPs are formed by a variety of operational groups, which are identified by FTIR (Fig 4). The FTIR analysis showed peaks at 3393 cm^-1^, 2920 cm^-1^, 1637 cm^-1, -1^1552 cm, 1407 cm^-1^, 1019 cm^-1^, 800 cm^-1^, and 643 cm^-1^, as seen in Fig 4. The O-H stretching is commonly observed in alcohols and phenols (3393 cm^-1^); C-H stretching is typically observed in alkanes or methyl groups (2920 cm^-1^); C = O stretching is frequently observed in ketones, esters, or aldehydes (1637 cm^-1^); and N-H bending is typically observed in primary amines or amides (1552 cm^-1^); 800 cm^-1^ indicates aromatic C-H out-of-plane bending in substituted benzene rings, while 643 cm^-1^indicates C-H out-of-plane bending in substituted benzene rings or alkene C-H bending. 1407 cm^-1^ suggests C-H bending in methyl groups or alkane deformation vibrations: 1019 cm^-1^indicates C-O stretching in ethers or secondary alcohols. By acting as fingerprints for certain functional groups within the molecule, these spectral peaks help identify and characterize the structure of the molecule via FTIR analysis. The study reported that bands at 3325 cm^-1^signified water presence akin to surface-bound OH bands, while 2330 and 2087 cm^-1^ indicated hydroxyl groups formed by water interacting with ZnO defects [42]. Additionally, a 1423 cm^-1^ band hinted at potential amide group presence, while the 1018 cm^-1^ peak confirmed ZnO existence. This implies that the coordination of water with ZnO defects produces hydroxyl species that can form additional bonds. The existence of the Zn–O combination was corroborated by peaks at 400–700 cm^-1^and 1018 cm^-1^, confirming the material’s structure. These results guarantee the purity of ZnONPs and allude to oxidation, which is important for comprehending its characteristics and wide range of uses.

#### 3.1.3. Scanning Electron Microscope (SEM) imaging.

**Fig 5:**
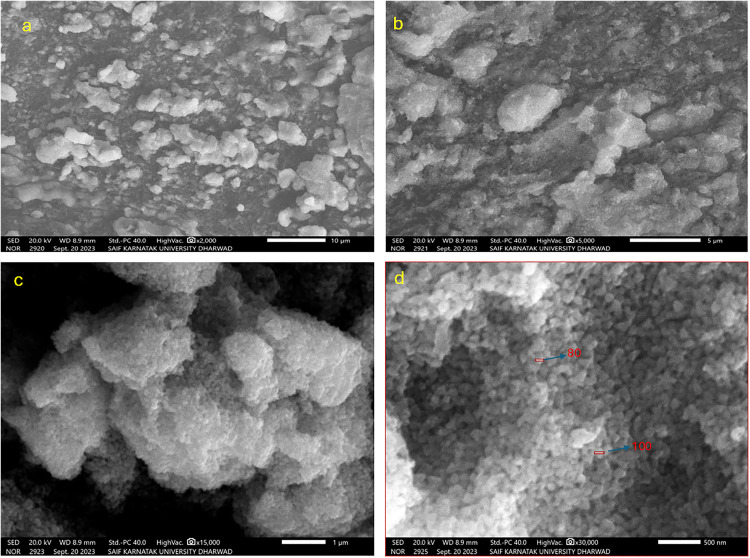
Scanning Electron Microscopy (SEM) images of zinc oxide nanoparticles (ZnONPs) synthesized using ginger peel extract. This figure presents SEM micrographs of ZnONPs captured at magnifications of (a) 2000 × , (b) 5000 × , (c) 15,000 × , and (d) 30,000 × . The images reveal the surface morphology and size distribution of the nanoparticles. The ZnONPs appear predominantly spherical, with some degree of agglomeration, indicating the presence of both individual nanoparticles and larger aggregates.

According to SEM analysis, the produced ZnONPs are spherical or agglomerated, as seen in the image (Fig 5). In a previous study, Vidya et al., reported the synthesis of spherical nanoparticles with diameters ranging from 11 to 25 nm [43]. SEM analysis revealed details about shape and size distribution of the synthesized materials, emphasizing the existence of both bigger aggregates and discrete nanoparticles in the sample.

#### 3.1.4. Energy Dispersive X-Ray (EDS) Spectroscopy.

**Fig 6:**
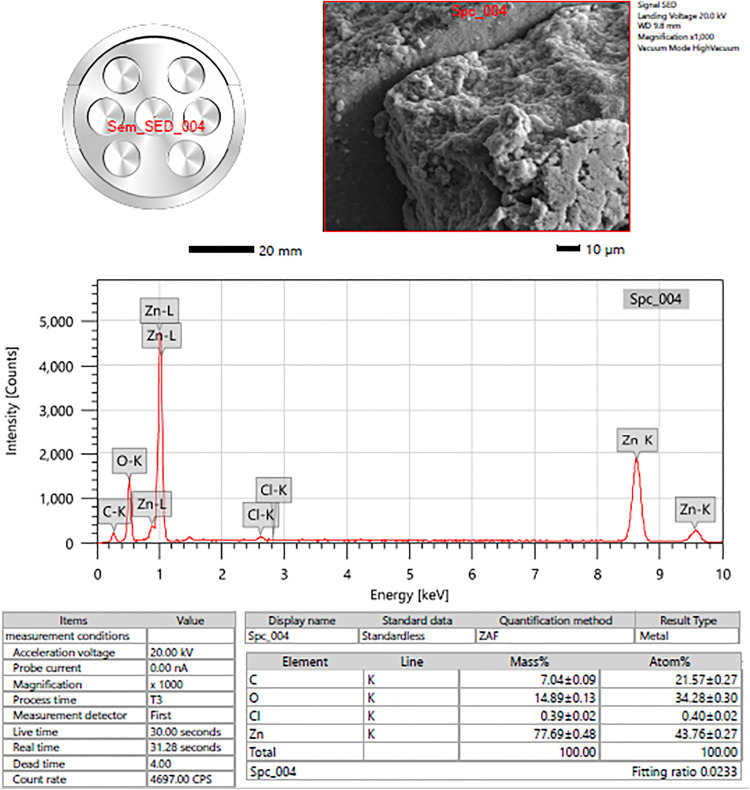
Scanning Electron Microscopy-Energy Dispersive X-ray Spectroscopy (SEM-EDS) analysis of zinc oxide nanoparticles (ZnONPs) synthesized using ginger peel extract. This figure shows the SEM-EDS image used to analyze the surface morphology and elemental composition of the synthesized ZnONPs. The EDS spectrum reveals prominent peaks corresponding to zinc (Zn), oxygen **(O)**, and carbon **(C)**, confirming the successful formation of ZnONPs. The quantitative analysis indicates that approximately 43.76% of the sample is composed of zinc oxide, validating the synthesis process. The presence of Zn and O aligns with expected ZnONPs composition, while carbon may originate from the plant extract used in the green synthesis.

The EDS spectra peaks corresponding to zinc (Zn), oxygen (O), and carbon (C), confirmed the successful formation of ZnONPs. Additionally, the EDS analysis indicated that 43.76% of the synthesized nanoparticles were composed of zinc oxide (Fig 6). EDS represents the percentage or amount of zinc oxide nanoparticles relative to the sample’s total composition. It validates the anticipated result of the synthesis process by confirming the successful creation or synthesis of zinc oxide nanoparticles as a substantial component of the studied material. A previous study reported that ZnO nanoparticles have a composition that is around 35% oxygen and 55% zinc [44]. This information validates the composition of the ZnO nanoparticles by confirming that the elemental components found match the predicted characteristics of the particles.

#### 3.1.5. XRD analysis to determine the characteristics of the ZnONPs.

**Fig 7:**
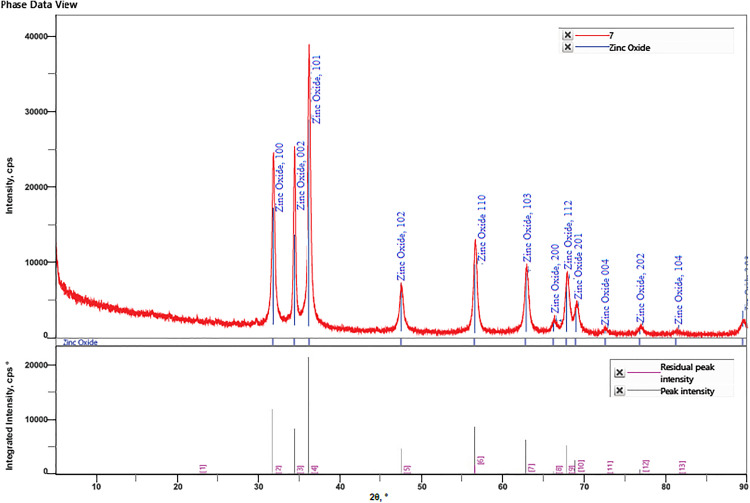
The X-ray Diffraction (XRD) pattern of ZnONPs, displaying distinct diffraction peaks corresponding to the spherical structure at 2θ values of 31.73°, 34.38°, 36.21°, 47.50°, 56.54°, 62.74°, and 68.92°, which correspond to specific lattice planes including (100), (002), (101), (102), (110), (103), (200), (112), (201), (004), (202), and (104). These peaks match the standard Zinc oxide pattern reported by the Joint Committee on Powder Diffraction Standards (JCPDS No. 36-1451), confirming the crystalline nature and phase purity of the nanoparticles.

The XRD analysis of ZnONPs synthesized using ginger peel extract revealed diffraction peaks at 31.73°, 34.38°, 36.21°, 47.50°, 56.54°, 62.74°, and 68.92°, corresponding to lattice planes (100), (002), (101), (102), (110), (103), (200), (112), (201), (004), (202), (104) (Fig 7, Table 1). These peaks confirm the spherical structure of ZnONPs, indicating high crystallinity and preferred orientations. The XRD pattern found is comparable to the Joint Committee on Powder Diffraction Standards (JCPDS) (NO 36–1451) and previous research [3,45,46], which are critical for their photocatalytic and biomedical applications. The findings align with Sekar Vijayakumar et al., who reported similar lattice planes for green-synthesized ZnONPs using plant extracts [3,45,46], validating the crystalline purity of the material. Comparatively, Janaki et al. observed identical peaks using ginger rhizome extract, though with broader peaks suggesting lower crystallinity, while Mongy & Shalaby reported sharper peaks with *Rhus coriaria* extract, indicating enhanced crystallinity due to optimized synthesis. The absence of extraneous peaks in our XRD pattern confirms the high purity of ginger peel-derived ZnONPs, surpassing impurity levels noted in some chemically synthesized ZnONPs by Zhang et al., 2010.

**Table 1:** Crystalline size from XRD data by Scherrer equation.

2 θ °	FWHM, °	Crystalline size D(mm)
22.49641	0.1234	65.63919258
31.73718	0.3655	22.59652384
34.38433	0.2474	33.61298023
36.21381	0.3784	22.08827034
47.50744	0.4888	17.7569077
56.54532	0.4307	20.9435575
62.74079	0.521	17.85874942
66.2593	0.3912	24.24959901
67.79269	0.5333	17.94652284
68.9209	0.5589	17.23936748
72.4508	0.3967	24.82449762
76.87857	0.5986	16.94370111
81.34798	0.551	19.01024746

Average = 24.670.

Recent findings by Benzarti et al. [47] further corroborate these observations. Their novel synthesis approach utilizing *Hylocereus undatus* cladode extracts yielded ZnONPs with comparable XRD peaks at 2θ values of approximately 31.5°, 34.4°, and 36.2°. Calcination at 400 °C was reported to enhance crystallinity; however, minor sodium and sulfur impurities were detected in their samples. In contrast, our synthesized ZnONPs exhibited a purer diffraction pattern, indicating higher phase purity and fewer elemental contaminants. Another study using *Calluna vulgaris* leaf extract confirmed a wurtzite structure with peaks at 31.78°, 34.42°, and 36.25°, showing improved crystallite size (35–40 nm) under optimized conditions, aligning with our findings of high crystalline quality. These comparisons underscore the robustness of ginger peel extract as an emerging green synthesis agent, enhancing NP stability and efficacy for antibacterial, antifungal, and anticancer applications.

The Debye-Scherrer Equation predicts the crystal size of ZnONPs. The Debye-Scherrer formula was used to determine the particle size of ZnONPs, and the result was 24.670 nm (Table 1). A study reported that the Scherrer formula indicates that the ZnO nanoparticles have an average grain size of about 24 nm [3].

The above Table 2 presents the phytochemical analysis of ginger peel extract, depicting the presence of alkaloids, saponins and steroids. Contrary to certain ginger peel reports, our phytochemical screening found no flavonoids or phenols. This is due to various factors that affect plant chemical makeup, including the variety, geographical origin, plant maturity, and, most importantly, extraction process and solvents used are all crucial. A recent study [48] identified terpenoids, carbohydrates, flavonoids, and alkaloids as the principal chemical constituents present in *Euphorbia hirta* leaf extract. Among these, phenolic compounds and flavonoids were found to play a pivotal role in the bioreduction, synthesis, and stabilization of metal and metal oxide nanoparticles, highlighting their significance in green nanotechnology approaches. In addition to polyphenols, other phytochemicals such as reducing sugars, polysaccharides, and alkaloids contribute significantly to the green synthesis of nanoparticles. Monosaccharides and polysaccharides act as both reducing and capping agents by donating electrons through the oxidation of their aldehyde groups, thereby facilitating the reduction of metal ions into stable nanoparticles. These biomolecules not only influence the nucleation and growth processes but also enhance the stability and biocompatibility of the synthesized nanomaterials.

**Table 2:** Preliminary phytochemicals screening results.

S. No	Tests	Results
1.	Alkaloids	+
2.	Flavonoids	–
3.	Saponins	+
4.	Steroids	+
5.	Triterpenoids	–
6.	Tannins	–
7.	Phenols	–
8.	Glycosides	–
9.	Anthraquinones	–
10.	Coumarins	–
11.	Diterpenes	–
12.	Catechin	–
13.	Anthocyanoside	–
14.	Resins	–
15.	Volatile Oils	–

*“+” indicate positive results and “–” indicate negative results.*

### 3.2. Optimization of ZnONPs synthesis parameters

#### 3.2.1. Effect of Temperature and pH.

The UV spectroscopy results (Table 3) for the ZnONPs produced at 30°C to 60°C revealed peaks at 368, 354, 364, and 364 nm. In a previous study, Awadh et al., reported that a rise in temperature causes the size of the nanoparticles to shrink [49]. It also causes the rate of reaction and the activation energy of the molecules to increase, resulting in the formation of smaller, monodispersed nanoparticles within the same volume. The narrow and sharp Surface Plasmon Resonance (SPR) waves in the current investigation indicate that ZnONPs size decreases with temperature.

**Table 3:** UV–Vis spectroscopy results of ZnONPs synthesized at varying temperatures.

Temperature (°C)	Peak value of the spectrum	Absorbance
30	368	0.3673
40	354	0.3163
50	364	0.3020
60	364	0.2826

**Fig 8:**
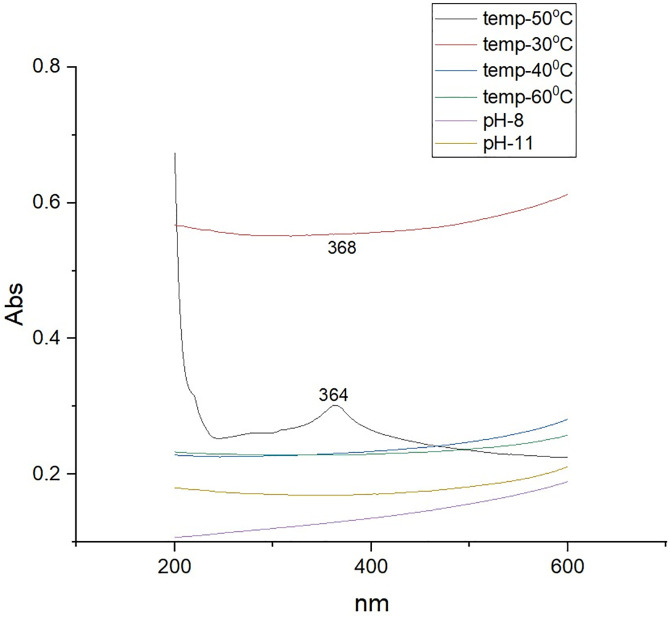
Ultraviolet-Visible (UV-Vis) spectral analysis illustrating the effect of temperature and pH on the ZnONPs. Peaks in the spectra represent Surface Plasmon Resonance (SPR), which correlates with nanoparticle size and dispersion. Optimal synthesis occurred at 50–60°C and pH 10, yielding stable ZnONPs with desirable optical properties.

In the current study, the peak was not visible at pH 6, 8 and 11 but it was visible at pH 10 at 364 nm. In a previous study, Bachir Gherb et al., revealed that the UV-Vis spectra showed a notable absorbance peak at 378 nm at pH 6 and pH 11. At pH 4, there was a peak visible at 350 nm, and at pH 9.5, there was a narrow peak visible at 378 nm [50]. Additionally, as the pH increased, the peak’s sharpness increased (Table 4, Fig 8). These methods streamline the investigation of ZnONPs by investigating their dimensions, geometries, composition, surface chemistry, and surface modification in addition to their crystal orientation, phases, cost-effectiveness, environmental friendliness [51–54].

**Table 4:** UV-Vis spectra analysis depicting the effect of pH on the synthesis of ZnONPs.

pH	Peak value of the spectrum	Absorbance
6	–	–
8	–	–
10	364	0.3020
11	–	–

### 3.3. Biomedical applications

#### 3.3.1. Anticancer activity against breast cancer MDA-MB-231 cell lines.

Zinc nanoparticles can be synthesized from various sources, including plant extracts. Plant extracts contain phytochemicals that can act as reducing agents and stabilizers during nanoparticle synthesis. These extracts often include compounds like flavonoids, phenols, terpenoids, and alkaloids, which can play a role in reducing zinc ions to nanoparticles and preventing their aggregation. Many studies have reported that zinc nanoparticles can inhibit the viability and proliferation of breast cancer cells in laboratory settings. The nanoparticles are believed to induce cytotoxic effects on cancer cells while sparing normal cells to some extent. This could potentially be attributed to their ability to generate ROS and cause oxidative stress, leading to cellular damage and apoptosis in cancer cells [55–58].

**Fig 9:**
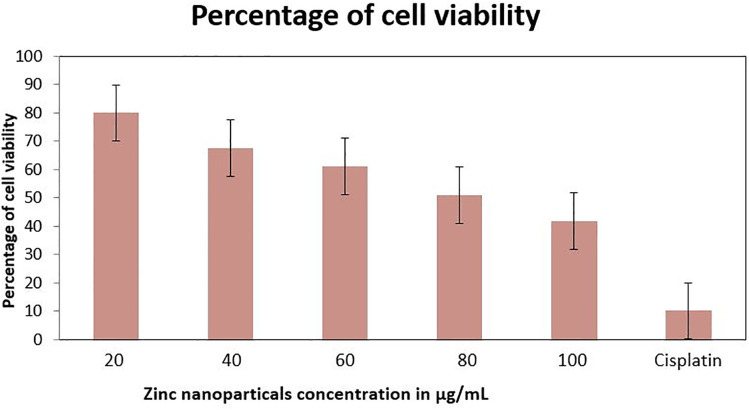
Percentage of cell viability of MDA-MB-231 breast cancer cells treated by ZnONPs. The cell viability decreased with increasing concentration of ZnONPs.

**Fig 10:**
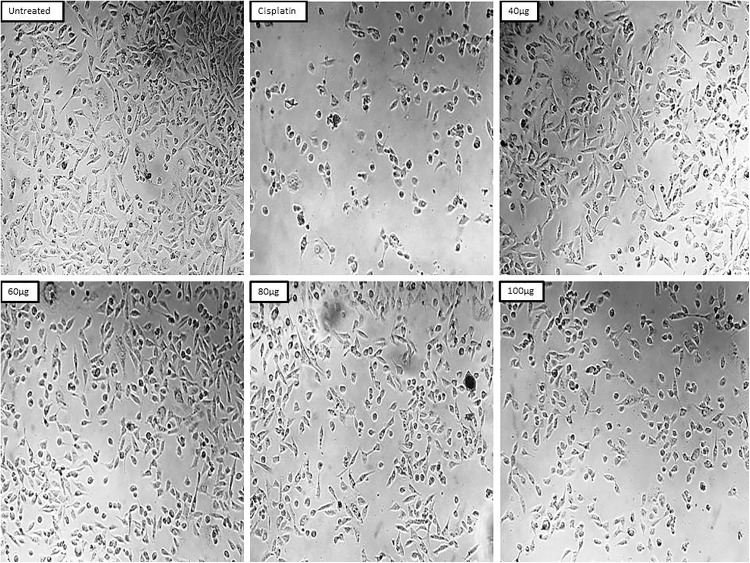
Representative micrographs showing morphological changes in breast cancer cells (MDA-MB-231) following treatment with biogenic zinc oxide nanoparticles (ZnONPs). This figure illustrates the cellular morphology of MDA-MB-231 cells after exposure to ZnONPs. Cells were treated with increasing concentrations of ZnONPs: 20, 40, 60, 80, and 100 µg/mL. The micrograph for 20 µg/mL is not included due to negligible morphological changes and technical constraints during imaging. Observable changes include cell shrinkage, elongation, detachment, and loss of turgidity, which are indicative of cytotoxic effects and reduced cell viability.

In the present study the anticancer activity of ZnONPs synthesized from the aqueous extract of *Z. officinale* peel was evaluated against breast cancer MDA-MB-231 cells by using standard procedure of MTT cell viability assay along with standard drug cisplatin and untreated group of cells as positive and negative control, respectively. The results revealed that the anticancer activity of zinc nanoparticles was seen in a dose dependent manner, i.e., with increasing concentration the percentage of cell viability decreased. At an initial concentration of 20 µg/mL, the percentage of cell viability was observed to be 79.97 ± 0.0028, and at its higher concentration 100 µg the percentage of cell viability decreased to 41.85 ± 0.0070 (Table 5 and Fig 9). The IC_50_ concentration was found to be 82.13 µg/mL. In the case of standard drug cisplatin, the percentage of cell viability was observed to be 10.08 ± 0.0042. Microscopic study revealed that in treated group of cells there is detachment of cells, cell turgidity, cell shrinkage and cell elongation. As the concentration of test sample was increased there were morphological changes in the cells (Fig 10). ZnONPs demonstrated notable anticancer activity against the MDA-MB-231 breast cancer cell line, suggesting their potential to induce cytotoxicity and suppress tumor cell proliferation.

**Table 5:** Percentage of cell viability of MDA-MB-231 breast cancer cells treated by ZnONPs and Cisplatin.

Sl. No	Treatments	Concentrations in µg/mL	Percentage of cell viability
1	Zinc nanoparticles	20	79.97 ± 0.0028
		40	67.67 ± 0.0049
		60	61.18 ± 0.0028
		80	50.82 ± 0.0063
		100	41.85 ± 0.0070
2	Cisplatin	15	10.08 ± 0.0042

The results are expressed as mean ± standard deviation.

#### 3.3.2. Antibacterial and antifungal activity.

**Fig 11:**
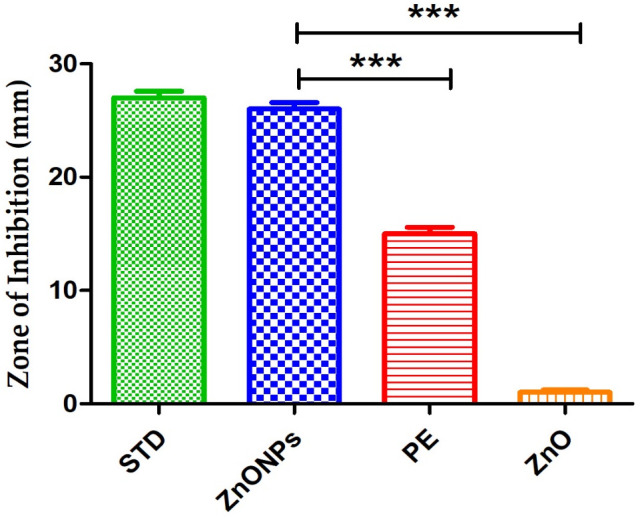
*Zingiber officinale* synthesized zinc oxide nanoparticles (ZnONPs) showed significant antibacterial activity against *Escherichia coli.* Data presented as mean ± SEM (n = 3). Statistical significance at ***p < 0.001 compared to Zinc oxide (ZnO).

**Fig 12:**
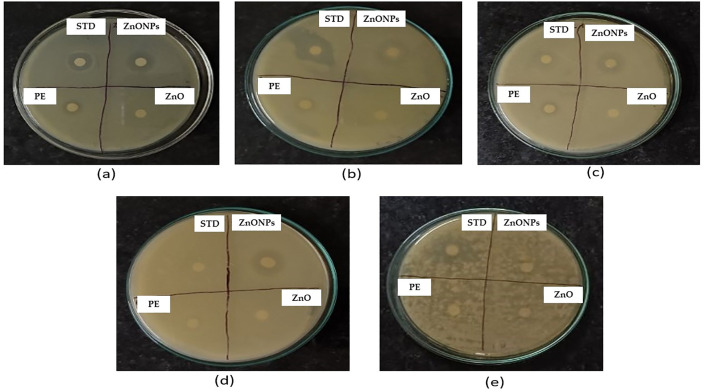
Antimicrobial activity of tested compounds against various bacterial strains. ***(a)*** **Escherichia coli (*ATCC no 25922)*, **(b)***Pseudomonas aeruginosa (ATCC no 27853)*, **(c)** *Bacillus subtilis (ATCC no 122264)*, **(d)** *Zymomonas mobilis (ATCC no 31821)* and ***(e)*** *Staphylococcus aureus (ATCC no 25923).* **ATCC =** American Type Culture Collection; STD = Standard antibiotic; PE = Plant extract; ZnO = Zinc oxide.

The antimicrobial activity of ginger peel-derived ZnONPs was evaluated using the disk diffusion method, following standard protocols. The NPs exhibited significant antibacterial activity against both Gram-positive and Gram-negative bacteria that were obtained from National Collection of Industrial Microorganisms (NCIM) in Pune, India. Clear zones of inhibition were observed for *E. coli* (27 mm), *P. aeruginosa*, *B. subtilis*, and *Z. mobilis*. However, conflicting results were noted for *S. aureus*: initial tests showed no zone of inhibition [59], possibly due to experimental variability or NP aggregation, while subsequent tests revealed a 21 mm inhibition zone (Table 6 and Fig 11 and 12).

**Table 6:** *Z. officinale* synthesized ZnONPs showed significant antibacterial activity.

Organism	Standard (Ampicillin) 25 µg/mL (mm)	*Z. officinale* ZnONPs 100 µg/mL (mm)	*Z. officinale* aqueous extract 100 µg/mL (mm)	Zinc oxide 100 µg/mL (mm)
***E. coli*** (Gram Negative)	27 ± 0.3	26 ± 0.28***	15 ± 0.21***	1 ± 0.3
***P. aeruginosa*** (Gram Negative)	31 ± 0.21	29 ± 0.41***	12 ± 0.13***	2 ± 0.4
***B. subtilis*** (Gram Positive)	22 ± 0.22	24 ± 0.43***	13 ± 0.09***	1 ± 0.18
***Z. mobilis*** (Gram Negative)	4 ± 0.24	21 ± 0.27***	11 ± 0.51***	1 ± 0.76
***S. aureus*** (Gram Positive)	21 ± 0.46	23 ± 0.38***	15 ± 0.21***	2 ± 0.41

Zone of inhibition Mean ± SEM for 3 readings. Statistical significance at ***p < 0.001 compared to ZnO.

**Fig 13:**
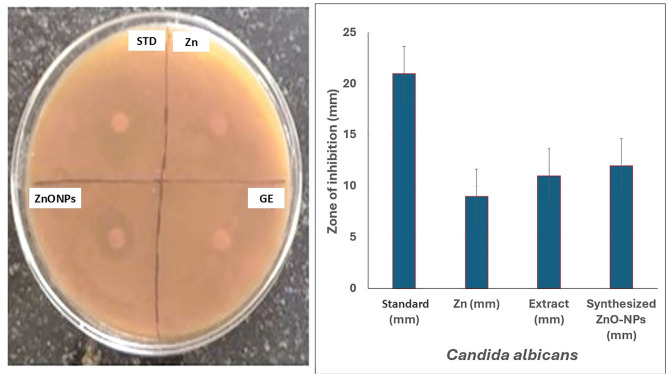
Antifungal activity depicting the zone of inhibition (ZOI). The ZOI measured 12 mm, indicating significant antifungal activity. STD- standard drug fluconazole; Zn- Zinc Acetate; GE-Ginger peel extract; ZnONPs -Zinc oxide nanoparticles.

The antifungal activity was assessed against *C. albicans*, a common opportunistic pathogen, resulting in a 12 mm zone of inhibition (Table 7, Fig 13). While Fluconazole resistance is possible in *C. albicans*, it’s generally less common and occurs at lower rates compared to other *Candida* species like *C. glabrata* and especially the highly concerning *C. auris*, underscoring the potential of ZnONPs as an alternative therapeutic agent against these pathogens. Given the increasing reports of multidrug resistance in *Candida* species, including *C. glabrata* and *C. auris*, it is crucial to find new treatments, and nanotechnology offers a promising solution, [60,61] as demonstrated by the inhibitory capacity of biosynthesized copper oxide nanoparticles against *C. albicans* through the production of ROS [62,63].

**Table 7:** Antifungal activity of ZnONPs against **C. albicans*.*

Microbial strains	Antibiotic (mm)	Zn (mm)	Extract(mm)	Synthesised ZnONPs (mm)
*C. albicans*	21 ± 0.3	9 ± 0.3	11 ± 0.3	12 ± 1.3

The antibacterial and antifungal effects of ZnONPs can be attributed to ROS generation and membrane disruption. ROS, such as superoxide and hydroxyl radicals, induce oxidative stress, damaging bacterial and fungal cell membranes and leading to cell lysis [64,65]. The positive surface charge of ZnONPs facilitates electrostatic interactions with negatively charged microbial membranes, causing physical disruption and leakage of cellular contents [60]. These mechanisms enhance the NPs’ efficacy against pathogens, including drug-resistant strains, supporting their potential in biomedical applications [40].

## 4. Conclusions

The current study demonstrates the successful green synthesis of ZnONPs using ginger peel extract, resulting in an environmentally friendly and cost-effective approach to nanoparticle production. The fabrication of stable zinc oxide nanoparticles with a spherical form and an average size of 24.67 nm was confirmed through extensive characterization. The biosynthesized nanoparticles showed significant antibacterial and antifungal activity against a wide range of pathogens. In addition, they exhibited potent anticancer activity against MDA-MB-231 breast cancer cells (IC_50_ = 82.13 μg/mL), which is comparable to the conventional drug cisplatin. This study’s findings offer a novel way to environmentally friendly production of nanoparticles. The findings underscore the potential of agro-waste-derived phytochemicals in nanoparticle synthesis, paving the way for scalable, eco-friendly nanotechnological applications. Furthermore, the study reveals the significant potential of ZnONPs derived from ginger peel as a natural and effective therapeutic agent for a wide range of biomedical applications, particularly in oncology and infectious disease treatment. To fully achieve their therapeutic potential and ensure their safety, further refinement of the synthesis process parameters is required, as well as in-depth in vivo studies are warranted.

### Limitations

This study evaluated the antifungal activity of ginger peel-derived ZnONPs against *C. albicans* only, which restricts the scope of the antifungal assessment. A broader spectrum analysis involving additional pathogenic fungi is warranted to enhance the biomedical relevance of these nanoparticles. Furthermore, cytotoxicity was assessed exclusively on the MDA-MB-231 breast cancer cell line without comparison to a non-cancerous cell line. This limits the evaluation of biocompatibility and selectivity index. Future investigations will include both normal cell lines and diverse fungal strains to provide a more comprehensive safety and efficacy profile.

## References

[1] Biswas P, Wu C-Y. Nanoparticles and the environment. J Air Waste Manag Assoc. 2005;55(6):708–46. doi: 10.1080/10473289.2005.10464656 16022411

[2] Chikkanna MM, Neelagund SE, Rajashekarappa KK. Green synthesis of zinc oxide nanoparticles (ZnONPs) and their biological activity. SN Appl Sci. 2019;1:1–10.

[3] Vijayakumar S, Vaseeharan B, Malaikozhundan B, Shobiya M. Laurus nobilis leaf extract mediated green synthesis of ZnO nanoparticles: characterization and biomedical applications. Biomed Pharm. 2016;84:1213–22. doi: 10.1016/j.biopha.2016.10.038 27788479

[4] Aldeen TS, Ahmed Mohamed HE, Maaza M. ZnO nanoparticles prepared via a green synthesis approach: physical properties, photocatalytic and antibacterial activity. J Phy Chem Solids. 2022;160:110313. doi: 10.1016/j.jpcs.2021.110313

[5] Li W, Qiu Z, Ma Y, Zhang B, Li L, Li Q, et al. Preparation and characterization of ginger peel polysaccharide-Zn (II) complexes and evaluation of anti-inflammatory activity. Antioxidants (Basel). 2022;11(12):2331. doi: 10.3390/antiox11122331 36552539 PMC9774354

[6] Das S, Das AK, Manda SC. Evaluation of antimicrobial activities of various solvent extracts of ginger rhizome peels and whole ginger rhizome without peels. J Pharm Res. 2017;6:1450–68.

[7] F R. Potato and Ginger Peels: a potential new source of natural antioxidants. MOJFPT. 2017;4(5). doi: 10.15406/mojfpt.2017.04.00103

[8] Ajayi OA, Oyerinde MO. Evaluation of nutritional composition of Roselle (Hibiscus sabdariffa) herbal tea infused with ginger (Zingiber officinale) and lemon (Citrus limon) peel. Aust J Sci Technol. 2020;4(1):215–21.

[9] Ali BH, Blunden G, Tanira MO, Nemmar A. Some phytochemical, pharmacological and toxicological properties of ginger (Zingiber officinale Roscoe): a review of recent research. Food Chem Toxicol. 2008;46(2):409–20. doi: 10.1016/j.fct.2007.09.085 17950516

[10] Janaki AC, Sailatha E, Gunasekaran S. Synthesis, characteristics and antimicrobial activity of ZnO nanoparticles. Spect Acta A Mol Biomol Spectrosc. 2015;144:17–22. doi: 10.1016/j.saa.2015.02.041 25748589

[11] Aliannezhadi M, Mirsanaee SZ, Jamali M, Shariatmadar Tehrani F. The physical properties and photocatalytic activities of green synthesized ZnO nanostructures using different ginger extract concentrations. Sci Rep. 2024;14(1):2035. doi: 10.1038/s41598-024-52455-z 38263199 PMC10807023

[12] Kebede Urge S, Tiruneh Dibaba S, Belay Gemta A. Green synthesis method of ZnO nanoparticles using extracts of zingiber officinale and garlic bulb (allium sativum) and their synergetic effect for antibacterial activities. J Nanomat. 2023;2023:1–9. doi: 10.1155/2023/7036247

[13] Radulescu D-M, Surdu V-A, Ficai A, Ficai D, Grumezescu A-M, Andronescu E. Green synthesis of metal and metal oxide nanoparticles: a review of the principles and biomedical applications. Int J Mol Sci. 2023;24(20):15397. doi: 10.3390/ijms242015397 37895077 PMC10607471

[14] Omran BA, Baek K-H. Valorization of agro-industrial biowaste to green nanomaterials for wastewater treatment: approaching green chemistry and circular economy principles. J Environ Manage. 2022;311:114806. doi: 10.1016/j.jenvman.2022.114806 35240500

[15] Sharma R, Lata S, Garg R. Valorisation of agricultural waste and their role in green synthesis of value-added nanoparticles. Environ Tech Rev. 2023;13(1):40–59. doi: 10.1080/21622515.2023.2283412

[16] Wu Z, Xia F, Lin R. Global burden of cancer and associated risk factors in 204 countries and territories, 1980-2021: a systematic analysis for the GBD 2021. J Hematol Oncol. 2024;17(1):119. doi: 10.1186/s13045-024-01640-8 39614359 PMC11607901

[17] Bray F, Laversanne M, Sung H, Ferlay J, Siegel RL, Soerjomataram I, et al. Global cancer statistics 2022: GLOBOCAN estimates of incidence and mortality worldwide for 36 cancers in 185 countries. CA Cancer J Clin. 2024;74(3):229–63. doi: 10.3322/caac.21834 38572751

[18] Vimala K, Sundarraj S, Paulpandi M, Vengatesan S, Kannan S. Green synthesized doxorubicin loaded zinc oxide nanoparticles regulates the Bax and Bcl-2 expression in breast and colon carcinoma. Process Biochem. 2014;49(1):160–72. doi: 10.1016/j.procbio.2013.10.007

[19] Mahdizadeh R, Homayouni-Tabrizi M, Neamati A, Seyedi SMR, Tavakkol Afshari HS. Green synthesized-zinc oxide nanoparticles, the strong apoptosis inducer as an exclusive antitumor agent in murine breast tumor model and human breast cancer cell lines (MCF7). J Cell Biochem. 2019;120(10):17984–93. doi: 10.1002/jcb.29065 31172567

[20] Mongy Y, Shalaby T. Green synthesis of zinc oxide nanoparticles using Rhus coriaria extract and their anticancer activity against triple-negative breast cancer cells. Sci Rep. 2024;14(1):13470. doi: 10.1038/s41598-024-63258-7 38866790 PMC11169510

[21] Subramaniam H, Djearamane S, Tey LH, Wong LS, Gupta PK, Janakiraman AK. Potential of zinc oxide nanoparticles as an anticancer agent: a review. J Exp Biol Agric Sci. 2022;10(3):494–501.

[22] Patil RB, Chougale AD. Analytical methods for the identification and characterization of silver nanoparticles: a brief review. Materials Today: Proceedings. 2021;47:5520–32. doi: 10.1016/j.matpr.2021.03.384

[23] Agarwal H, Nakara A, Shanmugam VK. Anti-inflammatory mechanism of various metal and metal oxide nanoparticles synthesized using plant extracts: a review. Biomed Pharmacother. 2019;109:2561–72. doi: 10.1016/j.biopha.2018.11.116 30551516

[24] Thakral F, Bhatia GK, Tuli HS, Sharma AK, Sood S. Zinc oxide nanoparticles: from biosynthesis, characterization, and optimization to synergistic antibacterial potential. Curr Pharmacol Rep. 2021;7:15–25.

[25] Darshita MN, Sood R. Review on synthesis and applications of zinc oxide nanoparticles. 2021;1–23.

[26] Hamza M, Muhammad S, Zahoor S. Biologically synthesized zinc oxide nanoparticles and its effect—a review. Acta Sci Appl Phys. 2022;2(9):3–10.

[27] Srivastava V, Gusain D, Sharma YC. Synthesis, characterization and application of zinc oxide nanoparticles (n-ZnO). Ceram Int. 2013;39(8):9803–8.

[28] Gaire BP, Lamichhane R, Sunar CB, Shilpakar A, Neupane S, Panta S. Phytochemical screening and analysis of antibacterial and antioxidant activity of Ficus auriculata (Lour.) Stem Bark. Pharmacognosy J. 2011;3(21):49–55. doi: 10.5530/pj.2011.21.8

[29] Christudas S, Kulathivel T, Agastian P. Phytochemical and antibacterial studies of leaves of Tridax procumbens L. Asian Pacific J Trop Biomed. 2012;2(1):S159–61. doi: 10.1016/s2221-1691(12)60149-x

[30] Edeoga HO, Okwu DE, Mbaebie BO. Phytochemical constituents of some Nigerian medicinal plants. Afr J Biotechnol. 2005;4(7):685–8.

[31] Onwukaeme DN, Ikuegbvweha TB, Asonye CC. Evaluation of phytochemical constituents, antibacterial activities and effect of exudate of Pycanthus angolensis Weld Warb (Myristicaceae) on corneal ulcers in rabbits. Trop J Pharm Res. 2007;6(2):725–30.

[32] Harborne AJ. Phytochemical methods: a guide to modern techniques of plant analysis. Springer Sci Bus Media; 1998. 1–295

[33] Mohammadi FM, Ghasemi N. Influence of temperature and concentration on biosynthesis and characterization of zinc oxide nanoparticles using cherry extract. J Nanostruct Chem. 2018;8:93–102.

[34] Takele E, Bogale RF, Shumi G, Kenasa G. Green synthesis, characterization, and antibacterial activity of CuO/ZnO nanocomposite using Zingiber officinale rhizome extract. J Chem. 2023;2023(1):3481389.

[35] Shaba EY, Jacob JO, Tijani JO, Suleiman MAT. A critical review of synthesis parameters affecting the properties of zinc oxide nanoparticle and its application in wastewater treatment. Appl Water Sci. 2021;11(2). doi: 10.1007/s13201-021-01370-z

[36] Wolf CR, Lewis AD, Carmichael J, Adams DJ, Allan SG, Ansell DJ. The role of glutathione in determining the response of normal and tumour cells to anticancer drugs. 1987;728–30. 10.1042/bst0150728 3678588

[37] Mosmann T. Rapid colorimetric assay for cellular growth and survival: application to proliferation and cytotoxicity assays. J Immunol Methods. 1983;65(1–2):55–63. doi: 10.1016/0022-1759(83)90303-4 6606682

[38] Hammer KA, Carson CF, Riley TV. Antimicrobial activity of essential oils and other plant extracts. J Appl Microbiol. 1999;86(6):985–90. doi: 10.1046/j.1365-2672.1999.00780.x 10438227

[39] Salih SJ, Smail AK. Synthesis, characterization and evaluation of antibacterial efficacy of zinc oxide nanoparticles. Pharm Biol Eval. 2016;3(3):327–33.

[40] Yassin MT, Mostafa AA-F, Al-Askar AA, Al-Otibi FO. Facile Green synthesis of silver nanoparticles using aqueous leaf extract of Origanum majorana with potential bioactivity against multidrug resistant bacterial strains. Crystals. 2022;12(5):603. doi: 10.3390/cryst12050603

[41] Shaban AS, Owda ME, Basuoni MM, Mousa MA, Radwan AA, Saleh AK. Punica granatum peel extract mediated green synthesis of zinc oxide nanoparticles: structure and evaluation of their biological applications. Biomass Conv Bioref. 2022;14(11):12265–81. doi: 10.1007/s13399-022-03185-7

[42] Rafique M, Tahir R, Gillani SSA, Tahir MB, Shakil M, Iqbal T, et al. Plant-mediated green synthesis of zinc oxide nanoparticles from Syzygium cumini for seed germination and wastewater purification. Int J Environ Anal Chem. 2022;102(1):23–38.

[43] Vidya C, Hiremath S, Chandraprabha MN, Antonyraj ML, Gopal IV, Jain A, et al. Green synthesis of ZnO nanoparticles by Calotropis gigantea. Int J Curr Eng Technol. 2013;1(1):118–20.

[44] Anbuvannan M, Ramesh M, Viruthagiri G, Shanmugam N, Kannadasan N. Synthesis, characterization and photocatalytic activity of ZnO nanoparticles prepared by biological method. Spectrochim Acta A Mol Biomol Spectrosc. 2015;143:304–8. doi: 10.1016/j.saa.2015.01.124 25756552

[45] Talam S, Karumuri SR, Gunnam N. Synthesis, characterization, and spectroscopic properties of ZnO nanoparticles. Int Sch Res Notices. 2012;2012(1):372505.

[46] Subramaniam H, Lim CK, Tey LH, Wong LS, Djearamane S. Oxidative stress-induced cytotoxicity of HCC2998 colon carcinoma cells by ZnO nanoparticles synthesized from Calophyllum teysmannii. Sci Rep. 2024;14(1):30198. doi: 10.1038/s41598-024-81384-0 39632962 PMC11618351

[47] Benzarti Z, Neiva J, Faia P, Silva E, Carvalho S, Devesa S. Novel green synthesis route of ZnO nanoparticles for dielectric applications. Nanomaterials (Basel). 2025;15(13):991. doi: 10.3390/nano15130991 40648698 PMC12251299

[48] Ahmad W, Kalra D. Green synthesis, characterization and anti microbial activities of ZnO nanoparticles using Euphorbia hirta leaf extract. J King Saud Univ Sci. 2020;32(4):2358–64. doi: 10.1016/j.jksus.2020.03.014

[49] Al Awadh AA, Shet AR, Patil LR, Shaikh IA, Alshahrani MM, Nadaf R, et al. Sustainable synthesis and characterization of zinc oxide nanoparticles using raphanus sativus extract and its biomedical applications. Crystals. 2022;12(8):1142. doi: 10.3390/cryst12081142

[50] Gherbi B, Laouini SE, Meneceur S, Bouafia A, Hemmami H, Tedjani ML, et al. Effect of pH value on the bandgap energy and particles size for biosynthesis of ZnO nanoparticles: efficiency for photocatalytic adsorption of methyl orange. Sustainability. 2022;14(18):11300. doi: 10.3390/su141811300

[51] Singh A, Kaushik M. Physicochemical investigations of zinc oxide nanoparticles synthesized from Azadirachta Indica (Neem) leaf extract and their interaction with Calf-Thymus DNA. Results in Physics. 2019;13:102168. doi: 10.1016/j.rinp.2019.102168

[52] Thema FT, Manikandan E, Dhlamini MS, Maaza MJ. Green synthesis of ZnO nanoparticles via Agathosma betulina natural extract. Mater Lett. 2015;161.

[53] Jafarirad S, Mehrabi M, Divband B, Kosari-Nasab M. Biofabrication of zinc oxide nanoparticles using fruit extract of Rosa canina and their toxic potential against bacteria: a mechanistic approach. Mater Sci Eng C Mater Biol Appl. 2016;59:296–302. doi: 10.1016/j.msec.2015.09.089 26652376

[54] Normah N, Juleanti N, Palapa NR. Hydrothermal carbonization of rambutan peel (Nephelium lappaceum L.) as a green and low-cost adsorbent for Fe(II) removal from aqueous solutions. Chem Ecol. 2022;38(3):284–300.

[55] Derakhshan F, Reis-Filho JS. Pathogenesis of triple-negative breast cancer. Annu Rev Pathol. 2022;17:181–204. doi: 10.1146/annurev-pathol-042420-093238 35073169 PMC9231507

[56] Hossain F, Majumder S, David J, Miele L. Precision medicine and triple-negative breast cancer: current landscape and future directions. Cancers (Basel). 2021;13(15):3739. doi: 10.3390/cancers13153739 34359640 PMC8345034

[57] Mohseni N, Sarvestani FS, Ardestani MS, Kazemi-Lomedasht F, Ghorbani M. Inhibitory effect of gold nanoparticles conjugated with interferon gamma and methionine on breast cancer cell line. Asian Pacific J Trop Biomed. 2016;6(2):173–8. doi: 10.1016/j.apjtb.2015.10.014

[58] Tian W, Wang C, Li D, Hou H. Novel anthraquinone compounds as anticancer agents and their potential mechanism. Future Med Chem. 2020;12(7):627–44. doi: 10.4155/fmc-2019-0322 32175770

[59] Albukhaty S, Al-Karagoly H, Dragh MA. Synthesis of zinc oxide nanoparticles and evaluated its activity against bacterial isolates. J Biotech Res. 2020;11:47–53.

[60] Arendrup MC, Patterson TF. Multidrug-resistant Candida: epidemiology, molecular mechanisms, and treatment. J Infect Dis. 2017;216(suppl_3):S445–51. doi: 10.1093/infdis/jix131 28911043

[61] Castro-Longoria E, Garibo-Ruiz D, Martínez-Castro S. Myconanotechnology to treat infectious diseases: a perspective. In: Fungal biology. Springer International Publishing; 2017. 235–61. doi: 10.1007/978-3-319-68424-6_12

[62] Garcia-Marin LE, Juarez-Moreno K, Vilchis-Nestor AR, Castro-Longoria E. Highly antifungal activity of biosynthesized copper oxide nanoparticles against Candida albicans. Nanomaterials (Basel). 2022;12(21):3856. doi: 10.3390/nano12213856 36364632 PMC9658237

[63] Zhang L, Jiang Y, Ding Y, Daskalakis N, Jeuken L, Povey M, et al. Mechanistic investigation into antibacterial behaviour of suspensions of ZnO nanoparticles against E. coli. J Nanopart Res. 2009;12(5):1625–36. doi: 10.1007/s11051-009-9711-1

[64] Sirelkhatim A, Mahmud S, Seeni A, Kaus NHM, Ann LC, Bakhori SKM, et al. Review on Zinc Oxide nanoparticles: antibacterial activity and toxicity mechanism. Nanomicro Lett. 2015;7(3):219–42. doi: 10.1007/s40820-015-0040-x 30464967 PMC6223899

[65] Chan YB, Aminuzzaman M, Chuah X-T, Li K, Balu P, Wong LS, et al. Review in green synthesis mechanisms, application, and future prospects for Garcinia mangostana L. (mangosteen)-derived nanoparticles. Nanotechnol Rev. 2025;14(1). doi: 10.1515/ntrev-2025-0157

